# Effect of alantolactones on cardiac parameters of animals under artificially induced oxidative stress

**DOI:** 10.37796/2211-8039.1457

**Published:** 2024-09-01

**Authors:** Mishal Fatima, Hina Andleeb, Tanzila Rehman, Ouz Gul, Shanza Azeezz, Huzaifa Rehman, Haq Nawaz

**Affiliations:** a Department of Biochemistry, Bahauddin Zakariya University, Multan, 60800, Pakistan; b Department of Chemistry, The Women University Multan, Multan, Pakistan

**Keywords:** Alantolactone, Oxidative stress, Antioxidant potential, *Inula helenium*, Free radical scavenging capacity, Cardiac biomarkers

## Abstract

**Purpose:**

Phytochemicals have been found effective in reducing the oxidative stress and damage to cardiovascular and other tissues. In this study, the effects of alantolactone (AL) on cardiac parameters in rabbits exposed to artificially-induced oxidative stress were investigated.

**Method:**

The oxidative stress was induced in a group of White New Zealand rabbits by injecting 40% hydrogen peroxide solution (1 ml/kg body weight) thrice with an interval of 72 h. The hydrogen peroxide-treated animals were orally treated with AL extracted from the roots of *Inula helenium* (1 ml/kg repeated thrice after 72 h). Blood samples were taken before and after the hydrogen peroxide and AL treatments, and the sera were subjected to analysis of oxidative damage in terms of malondialdehyde content (MDA), total antioxidant activity (TAOA), linoleic acid reduction capacity (LARC), hydroxyl radical scavenging capacity (HRSC), 2,2-diphenyl-1-picrylhydrazyl radical scavenging capacity (DPPH RSC), superoxide dismutase activity (SOD) and catalase activity, and cardiac parameters including troponin-I content (Trop-I), creatine kinase-MB (CKMB), aspartate transaminase (AST).

**Results:**

The hydrogen peroxide treatment substantially enhanced MDA content and SOD activity and decreased LARC, HRSC, DPPH, and catalase activity. The AL treatment significantly decreased MDA content, TAOA, Trop-I, CK-MB, and AST levels and increased LARC, DPPH RSC, HRSC, and catalase activity.

**Conclusion:**

The observed effect of AL treatment on the animals’ oxidative stress, antioxidant status, and cardiac biomarkers emphasizes that AL may effectively manage oxidative stress and cardiac damage.

## 1. Introduction

Heart failure, a chronic heart condition, is one of the main causes of death around the globe. Heart failure typically refers to the heart’s inability to maintain the blood flow required to meet the body’s metabolic needs. Cardiac remodeling is exclusively linked to the development of heart failure [1]. Importantly, remodeling is linked to poor outcomes, including an increased risk of ventricular dysfunction and cardiovascular mortality [2]. Hockam and Bulkley coined “cardiac remodeling” after observing regional dilation and thinning of infarcted myocardium in rats. It might be a maladaptive process or an adaptive one. In the first instance, structural alterations to the heart have a compensating impact that keeps the heart functioning normally.

On the other hand, during repeated stress, cardiac remodeling causes the heart to become gradually and irreversibly dysfunctional [3]. The deadliest adverse effects are systolic/diastolic left ventricular failure, which indicates weakened cardiac contraction/relaxation due to muscle thickening, tissue scarring, and decreased blood vessel density. Additionally, fibrosis results in electrical instability and unexpected cardiac death [4]. The available data supports the relationship between cardiac remodeling and the oxidative stress brought on by an increase in reactive species generation and a decrease in antioxidant defense. These effects include changes in calcium transport proteins, metalloproteinase activation, protein oxidation, disruption of DNA, enhanced fibroblast growth, cell death, and activation of hypertrophy signaling pathways [5]. As a result, myocardial remodeling appears to be significantly influenced by oxidative stress on a pathophysiological level.

Several pieces of evidence, particularly in animal models, have demonstrated that certain antioxidants can decrease the process of heart remodeling secondary to various traumas by lowering oxidative stress. Antioxidants may thus play a role in a therapy plan to treat this significant clinical disease [6]. A component extracted from the roots of *Inula racemose* called alantolactone (AL) displays various biological properties [7]. AL has been demonstrated to exhibit substantial activity against oxidative stress. This plant extract produces an impact by keeping glutathione levels in rats receiving isoproterenol treatment at normal levels. AL is effective in lowering lipid peroxidation and aids in maintaining healthy levels of glutathione. It exerts protective functions and aids in shielding the myocardium from the damaging effects of ROS. It has been discovered useful in protecting the heart from necrotic alterations brought on by myocardial ischemia [8]. In another study of two hundred patients with coronary heart disease, a 1:1 combination of *Commiphora mukul* and *I. racemose* was inquired. Chest pain was one of the main symptoms. Combining the plants *C. mukul* and *I. racemose* for pretreatment improvements in chest pain, the return of normal cardiac rhythms, and appreciable drops in cholesterol, triglycerides, and total lipid levels were all brought on by administering a 1:1 ratio to the patients.

Some European pharmacopeias have elecampane (*I. helenium*) on their official list of medicinal plants. The essential oil and extracts from *Inula helenium* L. roots are rich in lactones, mainly AL and isoalantolactone (IAL) [9].

Previously, it was reported that AL protects from myocardial ischemia by reducing oxidative stress. There has been little research on the direct effects of oxidative stress caused by hydrogen peroxide in rabbit models, even though the importance of oxidative stress in cardiovascular disorders is widely accepted. This study aims to inquire about the cardiovascular consequences of hydrogen peroxide-induced oxidative stress in live rabbits and the potential cardio-protective characteristics of AL. This research would significantly advance our understanding and be crucial in developing pharmaceutical medicine to reduce oxidative stress, hypertension, and myocardial ischemia, ultimately protecting against cardiac damage.

## 2. Material and methods

Different vendors were used to obtain chemicals and solvents DPPH, linoleic acid, ammonium thiocyanate, ammonium Molybdate, hydrochloric acid, hydrogen peroxide, Ethylenediaminetetraacetic acid (EDTA), iron chloride, and ethanol were given by Sigma–Aldrich Chemie GmbH (Taufkirchen, Germany). Salicylic acid, Sodium carbonate, sodium chloride, sodium phosphate, acetic acid (glacier), sulfuric acid, and methanol were provided by Merck Millipore (KGaA, Darmstadt, Germany). Sodium chloride and sodium hydroxide were given by Duksan Pharmaceutical Industrial Co. BDH Laboratory Supplies provided sulfuric acid, nitro blue tetrazolium (NBT), sodium phosphate, sodium carbonate, hypoxanthine, and MDA. Analyticalgrade chemicals and solvents were all used without further processing.

### 2.1. Study design

All procedures performed in studies involving animal participants were by the ethical standards of the institutional ethical research committee. The research examines whether treating rabbits with ALs can minimize oxidative stress and cardiovascular damage induced by H_2_O_2_. The experimental design involves causing oxidative stress through intramuscular delivery and regulating stress levels through response surface methods. AL obtained from *I. helenium* will be used to analyze the impact of H_2_O_2_ exposure. Blood samples will be taken from the rabbits to assess oxidative stress and specific biomarkers related to cardiovascular health. The collected data will be statistically evaluated to determine the effect of the treatment on these parameters. The therapeutic dosage will be adapted for subsequent administrations based on the suggested response surface model, providing optimized therapeutic effects.

### 2.2. Collection of plant samples and extraction of alantolactone

The dried *I. helenium* roots weighing 600g were available at the local herbal market. A recent study by Ref. [10] described a method to extract the desired components. The samples were ground to fine powder after being finely processed. A portion of this powder (100g) was treated for 2 h of reflux at 100 °C in 1000 ml of ethanol. The resultant solution was then entirely evaporated under reduced pressure, yielding a dark brown extract. The residual solution was then thoroughly combined with diethyl ether. The mixture was violently mixed before being transferred to a separating funnel. The separating funnel was placed carefully on a tripod platform and left alone overnight. As a result, two separate layers formed. After carefully draining the bottom layer, the topmost layer containing diethyl ether, AL was separated and placed in a beaker. The diethyl ether was vaporized and left a brown residue of AL (5.350 ± 0.2/100g). This residue was stored in a Petri dish to use later on.

### 2.3. Animals

Rabbits were chosen as experimental models because of their close phylogenetic relationship with humans, ease of handling, and huge blood volume. The local market provided ten male albino rabbits of the White New Zealand breed (weight: 1.00 0.20 kg). All of the rabbits used in the study were healthy, with no heart injury or anomalies symptoms. Each group contained five rabbits, the bare minimum for duplicate studies to minimize unnecessary animal torture.

Rabbits had free access to fresh water and were fed three meals daily, including grains and beans.

The rabbits were permitted to roam freely in a natural environment after the experiments.

### 2.4. Induction of oxidative stress

An initial experiment was conducted with five rabbits to determine the best hydrogen peroxide dosage. To make a 10 ml solution, dilute 2 ml of hydrogen peroxide with 8 ml of pure water, resulting in a 20% concentration. Additional concentrations were determined by individually diluting 4 ml, 6 ml, 8 ml, and 10 ml of hydrogen peroxide with distilled water. The rabbits were given varied amounts of these solutions, with those given 2 ml and 4 ml surviving, while those given higher quantities did not survive. A new set of rabbits was employed in a subsequent experiment. The hydrogen peroxide stock solution was diluted 2:3 with distilled water. The resultant solution was then diluted separately with distilled water to make 10 ml solutions with doses of 2 ml, 4 ml, 6 ml, 8 ml, and 10 ml. Surprisingly, all of the rabbits in this experiment survived, regardless of the diluted fluid they were given. Following the optimization procedure, ten rabbits were split into two groups. Every 72 h for ten days, both groups received 1 ml/kg intramuscularly injected hydrogen peroxide combined with distilled water (2:3) to create oxidative damage in the heart.

### 2.5. Treatment with AL

Blood samples were taken following oxidative stress induction, and a 24 h delay was allowed before providing the therapeutic dosage. The animals in group one were given AL dissolved in ethanol orally every 72 h for 10 days at a 1 ml/kg dose, while the other group was left untreated to study typical physiological fluctuations. The animals’ welfare and ethical considerations were prioritized throughout the inquiry. The research team strictly followed the rules established by the Committee of Medical Ethics for Animal Experiments, assuring proper animal handling, accurate therapy delivery, and post-treatment care.

### 2.6. Blood sampling

The blood samples were collected 24 h before and after hydrogen peroxide and AL treatments using a sterile 22 gauge, 1.5-inch long syringe needle into vacuum tubes containing gel and clot activator (Y330984 IMPROVACUTERR, Guangzhou Improve Medical Instruments, China). The sera were obtained by centrifugation of blood samples for 20 min at 4000×*g* in Eppendorf tubes and preserved at −20 °C, followed by oxidative stress, antioxidant potential, and cardiac biomarkers analysis.

### 2.7. Oxidative stress and antioxidant potential

#### 2.7.1. Malondialdehyde content

The oxidative stress was determined in terms of the malondialdehyde content of the study group. A 100 μL blood sample was treated with a solution containing 46 mM thiobarbituric acid (260 mg) in a 2.5 ml volume. This solution was made with 40 ml of 99% glacial acetic acid. The mixture was heated in a water bath for 35 min before being cooled to room temperature (25 ± 3 °C). The optical density of the MDA-TBA adduct at 562 nm was examined to assess malondialdehyde concentration [11].

#### 2.7.2. DPPH radical scavenging capacity

The RSC of DPPH was determined using a well-established method [12]. In this approach, a serum sample (100 μl) was added to a methanol-prepared 40M DPPH solution (3 ml) and left in the dark for 30 min. The DPPH solution alone was used as a control. The absorbance at 517 nm was measured, and the DPPH RSC was calculated using the following formula:


$$\text{DPPH RSC }(\%)=[({\text{Abs}}_{\text{control}}-{\text{Abs}}_{\text{sample}})/{\text{Abs}}_{\text{control}}]\times 100$$

#### 2.7.3. Linoleic acid reduction capacity

A well-established ferric thiocyanate method was used to evaluate the extracts’ Lipid Antioxidant Radical Capacity (LARC) [13]. The serum sample was initially treated with a 2.5% linoleic acid solution in ethanol. Then, 0.05M sodium phosphate buffer (2 ml) and distilled water (2 ml) were added to the mixture. The solution was then incubated in the dark for 24 h at 40 °C. A portion of the reaction mixture (1 ml) was mixed with 10 ml of 75% aqueous methanol following the incubation period. Then, 1 ml of a FeCl_3_ solution (20 Mm) was introduced. 1 ml of a 30% ammonium thiocyanate solution was added to the mixture to start the reaction. The absorbance of the reaction mixture was noted at 500 nm against a blank (reaction mixture without FeCl_3_). A butylated hydroxyl toluene (BHT) solution was used as the standard for comparison, and a control sample without extract was included. The following equation was used to determine the LARC:


$$\text{LARC }(\%)={\text{Abs}}_{\text{control}}{-\text{Abs}}_{\text{sample}}/{\text{Abs}}_{\text{control}}\times 100$$

#### 2.7.4. Hydroxyl radical scavenging capacity

HRSC was determined using a modified technique. The sample was mixed with FeSO4 solution (9 Mm) and salicylic acid (9 mM) in 95% ethanol (1 ml). The process was begun by adding 0.8 mM H_2_O_2_ (1 ml) and incubated for 30 min at 37 °C in a dark environment. A control solution without the sample was created, as was a blank without H_2_O_2_. At 510 nm, the absorbance of the reaction mixture was measured. The absorbance values were used in the calculation of the HRSC. The formula for the analysis of the HRSC value is as follows:


$$\text{HRSC }(\%)=[1-\{({\text{Abs}}_{\text{sample}}-{\text{Abs}}_{\text{blank}}\;\text{sample blank})/{\text{Abs}}_{\text{control}}\}]\times 100$$

A solution of Butylated hydroxytoluene (BHT) was used as a standard [14].

#### 2.7.5. Total antioxidant activity

A 1:1:1 v/v mixture of sodium phosphate solution (28 mM), sulfuric acid (0.6 M), and ammonium Molybdate solution (4 mM) was made to assess TAOA using the phosphomolybdenum test [15]. The mixture (3 ml) and serum sample (100 μL) were incubated for 90 min at 95 °C. A spectrophotometer (UV–Visible 6405, Jenway, Japan) measured absorbance at 695 nm with a blank as a reference. TAOA was evaluated after cooling the reaction mixture to room temperature (25–5 °C) by estimating BHT equivalent total antioxidant activity (mg/100 ml) using a regression equation constructed from the BHT standard curve (R2 = 0.9811).


TAOA (mg/100 ml)=(Absorbance of sample)/0.0539

### 2.8. Antioxidant enzymes

#### 2.8.1. Superoxide dismutase

Serum samples (100 μL) were deposited in test tubes and supplemented with nitro blue tetrazolium (0.75 mM), hypoxanthine (3 Mm), and sodium carbonate buffer (50 mM). The reaction was started when 10 μL of xanthine oxidase solution (0.75 Mm) was added to each test tube. A spectrophotometer (UV–Visible 6405, Jenway, Japan) set at 560 nm was used to measure the absorbance of the reaction mixture at 25 °C [16].

#### 2.8.2. Catalase

A mixture of 2M hydrogen peroxide solution (0.4 ml) and 0.01M phosphate buffer (0.2 ml) at pH 7 were mixed serum sample (100 μL). An aliquot (1 ml) from the reaction mixture was mixed with dichromate acetic acid (5% potassium dichromate and 98% glacial acetic acid solution 1:3 ratio), followed by boiling for 10 min. The mixture was cooled to room temperature, and absorbance was noted at 570 nm [17].

### 2.9. Cardiac and liver function tests

The in vitro testing reagent kit was designed to measure CK-MB and Trop-I activity in serum quantitatively. It made it possible to precisely assess the enzyme’s activity in a serum sample while Aspartate aminotransferase (AST), also known as S-glutamate oxaloacetate transaminase (SGOT), was measured in serum using the AST assay kit.

### 2.10. Statistical analysis

The effect of the botanical intervention on oxidative stress and cardiac parameters was investigated in the current research by evaluating serum antioxidant levels before and after induction and treatment. The findings were presented as mean ± standard deviation of five independent replicates. A dose-dependent study of cardiac function enzymes was performed, and statistical analysis was completed. Tukey’s multiple range tests were used for additional statistical analysis after a one-way analysis of variance (ANOVA) was performed using SPSS version 23 software. These comprehensive methodologies aided in the identification and assessment of significant differences between treatment groups in terms of oxidative stress and cardiac remodeling.

## 3. Results

This study looked at the effectiveness of a AL, specifically alantolactone, in minimizing the risk of heart disease and oxidative damage in experimental rabbits. These plant-derived bioactive chemicals are renowned for their therapeutic activities. The study optimized the production of oxidative stress in rats with hydrogen peroxide while delivering AL therapies using response surface approaches. Blood and serum samples were analyzed to determine oxidative stress levels and biomarkers for cardiovascular disease. The findings revealed phyto-lactones’ medicinal potential in reducing oxidative stress and treating cardiovascular illnesses.

### 3.1. Extraction yield

Based on the dry weight, 5.350.2/100 g AL was obtained from *I. helenium* powder, which validates the effective isolation of these bioactive chemicals from plant material, showing their potential benefits and their applications in various sectors.

### 3.2. Physiological parameters

Behavioral indicators were analyzed to investigate the influence of oxidative stress on rabbits’ psychological well-being. Locomotor activity modifications, such as altered movement patterns and decreased exploratory behavior, underlined the impact of oxidative stress on physical activity levels. Anxietylike behavior suggested the existence of feelings of anxiousness induced by oxidative damage. Still, markers of depression-like behavior, such as less social involvement and lack of movement in behavioral tests, suggested potential detrimental consequences on emotional state.

### 3.3. Malondialdehyde content

**Fig. 1:**
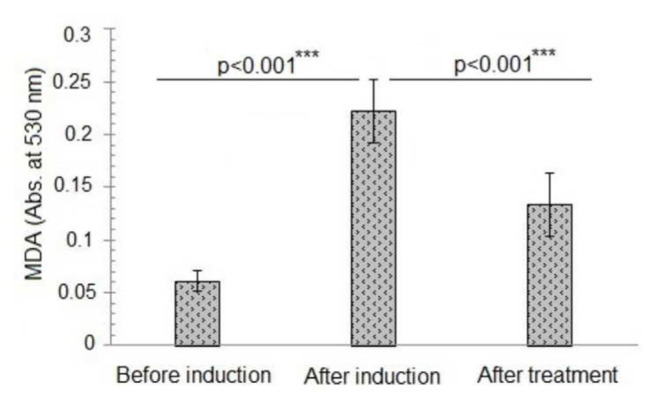
Malondialdehyde levels of different study groups before and after induction of oxidative stress and treatment with alantolactons. ***The p-value indicates the significant variation in malondialdehyde content of animals after induction of oxidative stress and alantolactone treatment at probability p < 0.001. The data is presented as mean ± standard deviation of three replicates. The means values of malondialdehyde content before and after induction of oxidative stress and after alantolactone treatment were compared at a 95% confidence level (p ≤ 0.05) by one-way analysis of variance (ANOVA) utilizing Duncan’s multiple range test in SPSS version 23.

Induction of oxidative stress significantly elevates MDA levels from 0.061 ± 0.01 to 0.222 ± 0.03 with a percentage increase of 72.2 ± 5.94%, as represented in (Figs. 1 and 6). MDA levels dropped to 0.134 ± 0.03 after treatment with a percentage of 32.2 ± 15.4%. Statistical analysis (p < 0.0001) verified the considerable difference in MDA levels between induction and treatment, suggesting successful oxidative stress reduction.

### 3.4. Antioxidant activity test

**Fig. 2:**
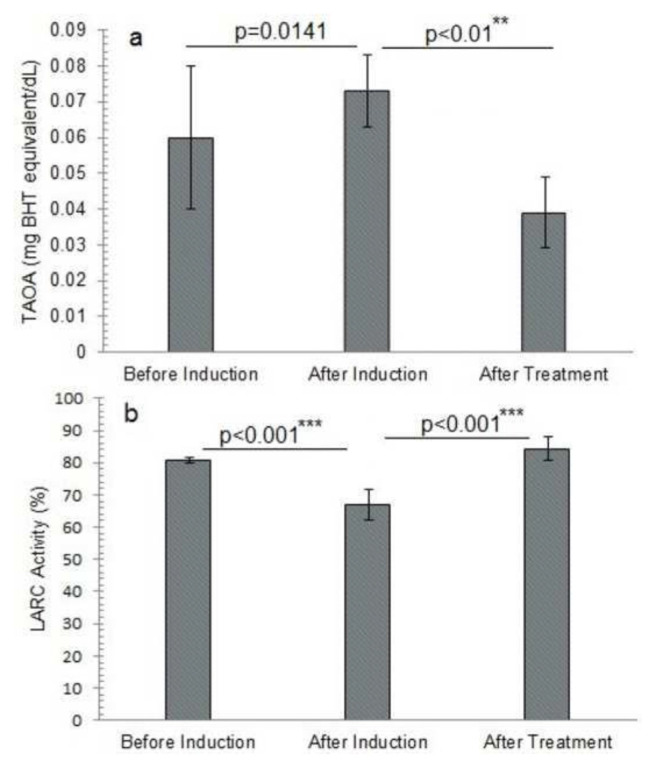
Antioxidant potential of different study groups before and after induction of oxidative stress and treatment with alantolactons. a) TAOA: Total antioxidant activity, b) LARC: Linoleic acid reduction capacity **The p-value indicates significant variation in total antioxidant activity of animals after alantolactone treatment at probability p < 0.01.***The p-value indicates the significant variation in total antioxidant activity and linoleic acid reduction capacity of alantolactone-treated animals after induction of oxidative stress at probability p < 0.001. The data is presented as mean ± standard deviation of three replicates. The means values of the studied parameters before and after induction of oxidative stress and after alantolactone treatment were compared at a 95% confidence level (p ≤ 0.05) by one-way analysis of variance (ANOVA) utilizing Duncan’s multiple range test in SPSS version 23.

The initial total antioxidant activity (TAOA) was 0.0600 ± 0.02 mg/dL, which increased to 0.0730 ± 0.010 mg/dL after induction with a percentage increase of 23 ± 20.2% but then declined to 0.0390 ± 0.01 mg/dL following treatment with a percentage decline of 47.8 ± 15.1%, showing a decrease in total antioxidant activity relative to the pre-induction level On the other hand, Table 1 represented that LARC values reduced dramatically from 80.665 ± 0.93 before induction to 66.96 ± 4.81% after induction with a percentage decline of 17 ± 6.2% and then increased significantly to 84.31 ± 3.7799% with a percentage increase of 20.6 ± 4.9% following treatment, showing better linoleic acid reduction ability as shown in (Figs. 2 and 6).

### 3.5. Analysis of radical scavenging capacity

**Fig. 3:**
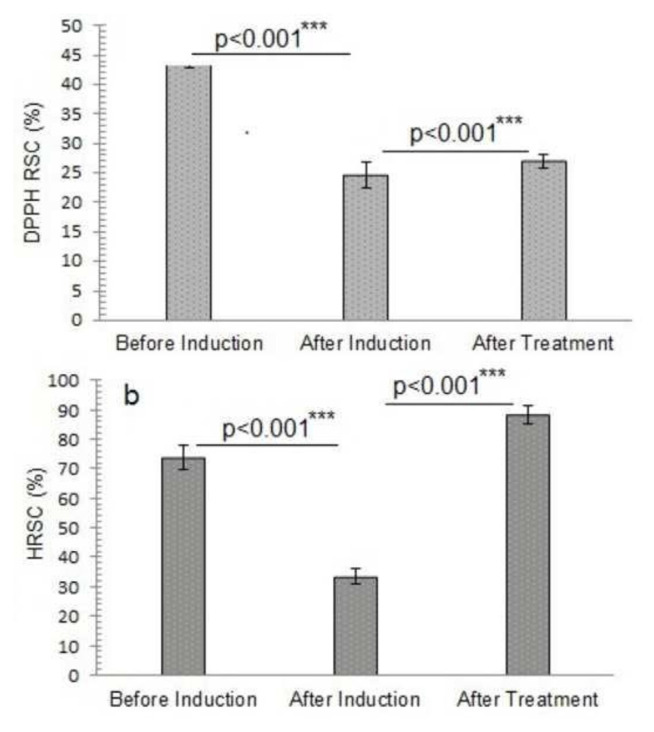
Free radical scavenging capacity of different study groups before and after induction of oxidative stress and treatment with alantolactons. a) DPPH RSC: 2, 2-Diphenyl-1-picrylhydrazyl radical scavenging capacity, b) HRSC: Hydroxyl radical scavenging capacity ***The p-value indicates the significant elevation in 2, 2-Diphenyl-1-picrylhydrazyl radical scavenging capacity and hydroxyl radical scavenging capacity of alantolactone-treated animals after induction of oxidative stress at probability p < 0.001. The data is presented as mean ± standard deviation of three replicates. The means values of the studied parameters before and after induction of oxidative stress and after alantolactone treatment were compared at a 95% confidence level (p ≤ 0.05) by one-way analysis of variance (ANOVA) utilizing Duncan’s multiple range test in SPSS version 23.

The radical scavenging capacity of the samples was also tested. The DPPH and HRSC test results revealed a baseline scavenging capacity of 44.303 ± 01.44 and 373.952 ± 19.16% before induction, which decreased dramatically to 24.550 ± 2.18, 33.520 ± 2.53% with a percentage decline of 44.4 ± 5.6, 54.6 ± 2.5% respectively after induction. After treatment, however, the DPPH and HRSC test scores climbed to 26.984 ± 1.26 and 442.135 ± 30.16%, with a percentage of 9.6 ± 5.1, 62 ± 2.1% respectively, showing increased antioxidant activity (Figs. 3 and 6). The ANOVA analysis yielded a p-value of 0.000, indicating a statistical difference between the DPPH and HRSC findings before and after therapy.

### 3.6. Analysis of enzymatic activity

**Fig. 4:**
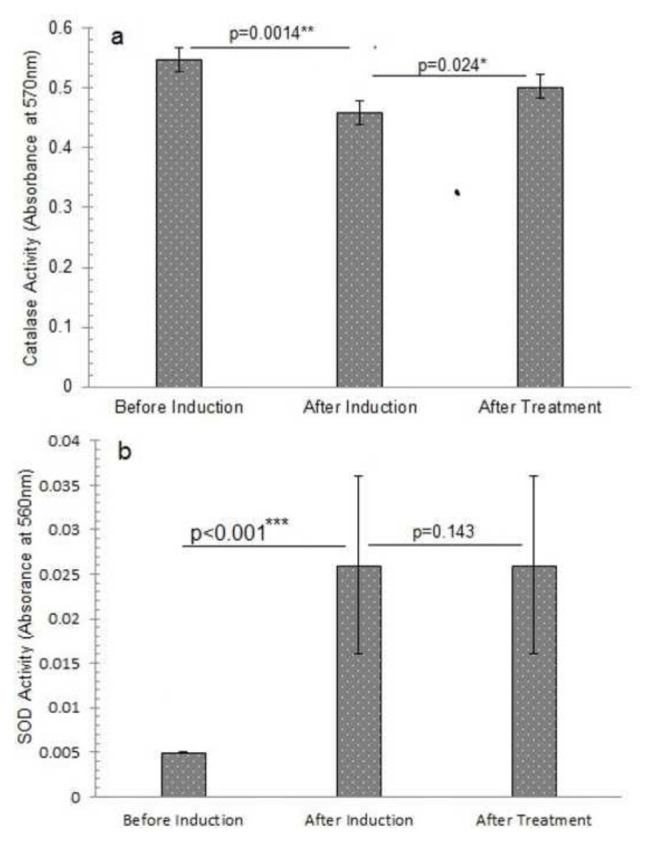
Antioxidant enzyme activity of different study groups before and after induction of oxidative stress and treatment with alantolactons. a) Catalase activity, b) Superoxide dismutase activity *The p-value indicates the significant elevation in Catalase activity of animals after alantolactone treatment at probability p < 0.05. **The p-value indicates the significant decline (p < 0.01) in Catalase activity of animals after induction of oxidative stress at probability p < 0.01. ***The p-value indicates the significant elevation in superoxide dismutase activity of alantolactone treated animals after induction of oxidative stress at probability p < 0.001. The data is presented as mean ± standard deviation of three replicates. The means values of the studied parameters before and after induction of oxidative stress and after alantolactone treatment were compared at a 95% confidence level (p ≤ 0.05) by one-way analysis of variance (ANOVA) utilizing Duncan’s multiple range test in SPSS version 23.

Tests were carried out to check the enzymatic activity of samples. The Catalase test revealed a value of 0.547 ± 0.04 absorbance at 570 nm before induction, which reduced to 0.459 ± 0.08 after oxidative stress induction with a percentage decline of 16.8 ± 15.6%. However, catalase activity went to 0.502 ± 0.02 after therapy with a percentage increase of 15.2 ± 11.2% (Figs. 4 and 6). It is indicated by the p-value (p-value >0.05) that the observed differences in Catalase activity before, during, and after treatment are unlikely to be statistically significant. SOD activity was 0.005 ± 0.00 absorbance at 560 nm before induction and 0.026 ± 0.01 absorbance at 560 nm after induction, showing a considerable increase with a percentage of 87 ± 8.1%. SOD test results, on the other hand, were constant at 0.026 ± 0.01 absorbance at 560 nm with a percentage of 26.8 ± 11.4% before and after treatment, indicating that the treatment had no obvious effect.

### 3.7. Cardiac and liver functioning tests

**Fig. 5:**
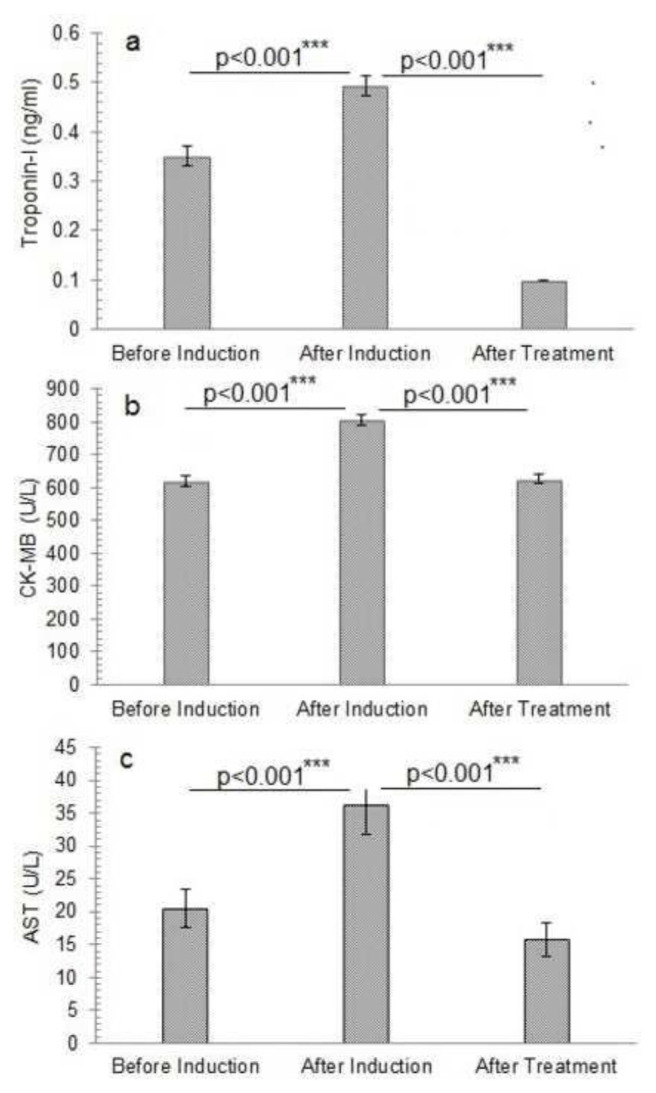
Caridac parameters of different study groups before and after induction of oxidative stress and treatment with alantolactons. a) Troponin-I level, b), Creatine kinase-MB level, c) Aspartate amino-transferase activity ***The p-value indicates the significant variation in Troponin-I and Creatine kinase-MB levels and aspartate aminotransferase activity of alantolactone-treated animals after induction of oxidative stress at probability p < 0.001. The data is presented as mean ± standard deviation of three replicates. The means values of the studied parameters before and after induction of oxidative stress and after alantolactone treatment were compared at a 95% confidence level (p ≤ 0.05) by one-way analysis of variance (ANOVA) utilizing Duncan’s multiple range test in SPSS version 23.

**Fig. 6:**
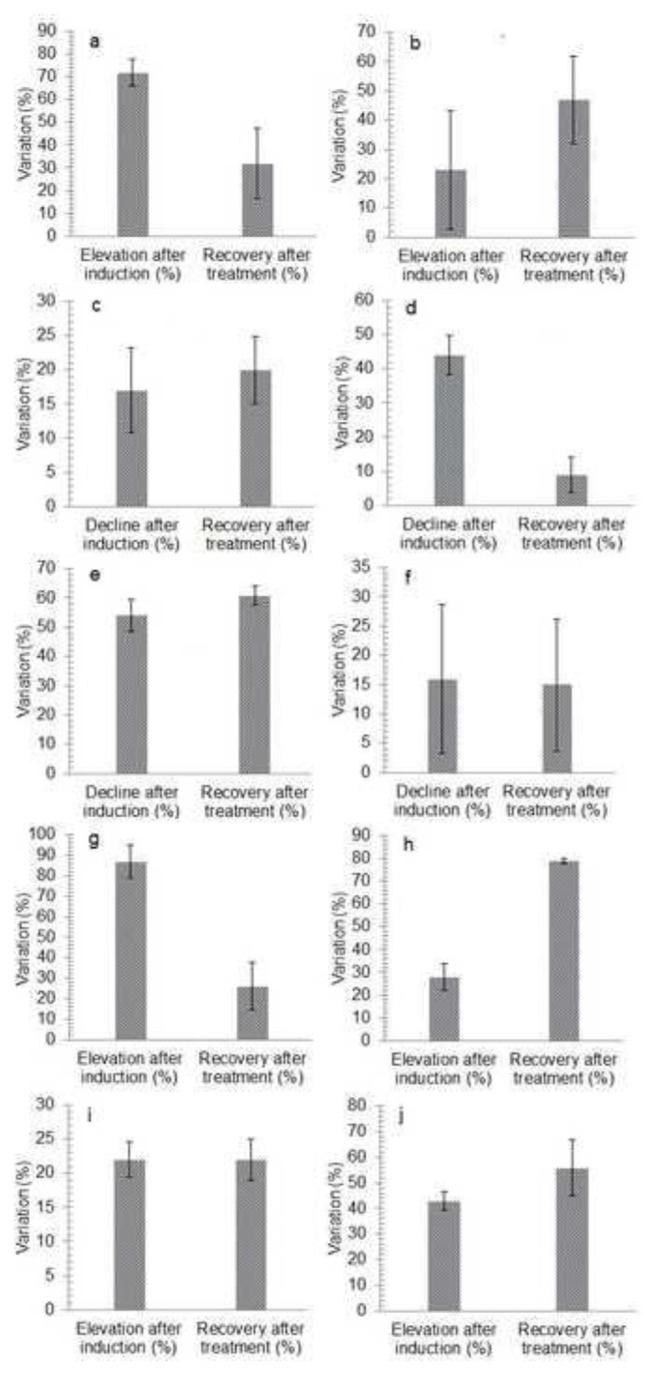
Percentage variation in oxidative stress and antioxidant potential and cardiac parameters of different study groups before and after induction of oxidative stress and treatment with alantolactons. a) Malondialdehyde (MDA) content, b) TAOA: Total antioxidant activity, c) LARC: Linoleic acid reduction capacity d) DPPH RSC: 2, 2 diphenyl–picrylhydrazyl radical scavenging capacity, e) HRSC: Hydroxyl radical ccavenging capacity f) Catalase activity g) SOD activity: Superoxide dismutase activity, h) Trop-I: Troponin-I level, i) CK-MB: Creatine myoglobin binding, j) AST: Aspartate aminotransferase.

Trop-I levels were 0.352 ± 0.02 ng/ml before induction, 0.494 ± 0.02 ng/ml after induction with a percentage increase of 28.2 ± 5.7%, and 0.100 ± 0.00 ng/ml following treatment with a percentage recovery of 79.2 ± 1.3%, as mentioned in. 0.000 was the observed p-value. The CKMB test revealed that the initial value was 620.160 ± 15.07 U/ L before induction, increased to 805.22 ± 16.22 U/L after induction with a percentage increase of 22.8 ± 2.5%, and fell to 625.30 ± 13.98 U/L with a percentage recovery of 22 ± 3.1% following treatment as shown. The AST test was performed using standard kits, and the results demonstrated levels of 20.440 ± 2.90 U/L before induction, 36.300 ± 4.54 U/L after induction of oxidative stress with a percentage elevation of 43.6 ± 3.7%, and 15.860 ± 2.54 U/L with a percentage of 55 ± 10.8% after therapy. The results of the above parameters are represented by Figs. 5 and 6.

## 4. Discussion

MDA content effectively indicates lipid peroxidation and the degree of oxidative damage. The oxidative stress was effectively generated in the test animals, as reported by the rise in MDA content and the significant p-value ( *p* = *0.000*) [18]. The results emphasize the importance of H_2_O_2_ induced oxidative stress paradigms for studying cardiovascular disease biology. Initially, greater MDA levels were observed, but subsequent treatment with antioxidant-rich AL effectively lowered MDA levels [19] indicating successful oxidative stress reduction and less oxidative damage.

The elevated TAOA value after induction suggests that antioxidant activity is upregulated in response to oxidative stress. However, changes in antioxidant compounds, affinities, and specificities and the dynamic nature of oxidative stress and antioxidant response may influence the decrease in TAOA activity following treatment. The duration and timing of the antioxidant treatment must be addressed for substantial improvements to occur. After H_2_O_2_ induction, the LARC value dropped from 80.665 ± 0.93 to 66.96 ± 4.81%, indicating a significant decrease in the sample’s capability to scavenge linoleic acid radicals. This reduction suggests that induction of H_2_O_2_ increased linoleic acid oxidation via free radical generation [20]. The following increase in LARC activity after treatment, on the other hand, suggests that the therapy strengthened antioxidant defense systems, improving the ability of the sample to neutralize linoleic acid radicals [21].

Upon induction, the significant drop in the DPPH reading shows that oxidative stress has damaged antioxidant protection mechanisms [22]. However, the ensuing increase in DPPH activity after treatment demonstrates the treatment’s efficacy in enhancing antioxidant activity [23]. This increase in the DPPH test value suggests good oxidative stress reduction and antioxidant capacity enhancement.

The HRSC value was adequate, but after induction, it dropped significantly from 373.952 ± 19.16 to 33.520 ± 2.53%, indicating poor hydroxyl radical neutralization. The results emphasize induction’s impact on the antioxidant protection system, reducing the ability to combat highly reactive hydroxyl radicals [24]. The subsequent increase in HRSC activity after therapy can be attributed to specific antioxidant compounds, which efficiently increase the sample’s capability to reduce hydroxyl radicals [25].

The induction reduced catalase activity, indicating a reduced ability to properly decompose hydrogen peroxide [26]. The catalase activity after induction yielded a value of 0.459 ± 0.08. However, after therapy, catalase activity improved nearly to 0.502 ± 0.02 abs. at 570 nm. The results suggest that the treatment may have reduced inhibitory chemicals or oxidative stress, enabling catalase to work at a greater rate [27].

Following induction, the increase in SOD activity can result from an oxidative stress induced biological response that stimulates SOD production to neutralize superoxide radicals and protect cells [28]. The treatment’s lack of effect on SOD activity suggests that the ALs’ protective effects may involve other mechanisms, such as direct scavenging of ROS or stimulation of other antioxidant defense mechanisms, which decrease oxidative damage without influencing SOD activity [29].

The increase in Trop-I and CK-MB values after induction indicates ventricular muscle distress or damage., which may be associated with diseases such as ischemia or myocardial infarction [30]. On the other hand, the fall in troponin-I and CK-MB levels following treatment implies that AL therapy is beneficial in avoiding additional cardiac injury. Interventions that enhance cardiac output reduce fibrosis, or promote regeneration of tissues could all have been attributed to the reported decrease in CK-MB concentrationsn, [31,32].

The rise in AST levels after induction suggests oxidative stress-induced damage to cardiac muscle cells, which could be caused by reduced blood circulation or the production of ROS. However, it is important to note that AST is not a specific marker for heart damage because it is also detected in various other organs [33]. A considerable decrease in AST concentrations after treatment indicates that the AL therapy successfully minimizes H_2_O_2-_induced heart damage caused by. The treatment may have lessened the unfavorable effects by boosting blood supply to the heart or promoting tissue repair, decreasing AST levels [34].

## 5. Conclusion

This study aimed to look at the potential benefits of AL in avoiding cardiac damage caused by oxidative stress. According to the findings, AL can lower oxidative stress indicators, increase antioxidant activity, and mitigate the detrimental effects of ROS on cardiac health. The results presented that ALs could be valuable medicinal products in treating oxidative stress-related cardiac disorders. The healthcare sector can look into developing novel medicines based on AL, expanding on the findings of this study and doing additional clinical tests and research. These encouraging developments pave the path for organic and efficient pharmaceutical therapy alternatives, providing consumers with new options while improving overall cardiac well-being.

## 6. Suggestion for future work

As reported in literature, many other related plants such as *Inula graveolens, Citrus limon*, and their compounds possessing good phytochemical profile, strong free radical scavenging activity against superoxide anion and hydroxyl radicals and high antioxidant potential [35,36], are also suggested be investigated for their cardioprotective potential.

## Figures and Tables

**Table 1:** Experimental value of parameters of oxidative stress, antioxidant potential and cardiac biomarkers before and after induction of oxidative stress and after alantolacton treatment in different study groups.

Parameters	Before Induction	After Induction	Variation (%)	p-value	After Treatment	Recovery (%)	p-value
MDA (Abs. at 530 nm)	*0.06±0.01	0.22±0.03	72.4±5.94	<0.0001	0.14±0.03	32.2±15.4	<0.0001
TAOA (mg/Dl)	0.06±0.02	0.07±0.01	23±2.20	0.141	0.04±0.01	47.8±15.1	0.002
LARC (%)	80.67±0.93	66.96±4.81	17±6.2	<0.0001	84.31±3.77	20.6±4.9	<0.0001
DPPH RSC (%)	44.30±1.44	24.55±2.18	44.4±5.6	<0.0001	26.99±1.26	9.6±5.1	<0.0001
HRSC (%)	73.95±4.16	33.52±2.53	54.6±2.5	<0.0001	88.14±3.16	62±2.1	<0.0001
Catalase activity	0.55±0.04	0.46±0.08	16.8±15.6	0.0014	0.50±0.02	15.2±11.2	0.024
SOD activity	0.01±0.00	0.05±0.01	80.10±6.1	<0.0001	0.026±0.01	13.33±1.4	0.143
Trop-I (ng/ml)	0.35±0.02	0.49±0.02	28.2±5.7	<0.0001	0.10±0.00	79.2±1.3	<0.0001
CK-MB (U/L)	620.16±15.07	805.22±16.22	22.8±2.5	<0.0001	625.30±13.98	22±3.1	0<.0001
AST (U/L)	20.44±2.90	36.30±4.54	43.6±3.7	<0.0001	15.86±2.54	55±10.8	<0.0001

MDA: Malondialdehyde, TAOA: Total antioxidant activity, LARC: Linoleic acid reduction capacity, DPPH: 2, 2-Diphenyl-1-picrylhy-drazyl, RSC: Radical scavenging capacity, HRSC: Hydroxyl Radical Scavenging Capacity, SOD: Superoxide dismutase, Trop-I: Troponin-I, CK-MB: Creatine myoglobin binding, AST: Aspartate Aminotransferase

* The data is presented as mean± standard deviation (SD) of five replicates. Based on the one-way analysis of variance (ANOVA), using Duncan’s multiple range test in SPSS version 23, the means with p≤0.05 are statistically similar at a 95% confidence level.

## Data Availability

The data associated with this study are openly available on the Mendeley data repository: https://data.mendeley.com/v1/datasets/publish-confirmation/std5pmbgx4/1.
